# Calyceal diverticulum masquerading as gross hydronephrosis: A diagnostic challenge

**DOI:** 10.1016/j.radcr.2026.05.077

**Published:** 2026-06-30

**Authors:** Komal Kumari Navik, Gaurav Raj, Roopal Malik

**Affiliations:** Department of Radiodiagnosis, Dr. Ram Manohar Lohia Institute of medical sciences, Lucknow, Uttar Pradesh, India

**Keywords:** Diverticulum, Hydronephrosis, CT urography, Delayed excretory phase

## Abstract

Calyceal diverticula are infrequent congenital anomalies of the renal collecting system that often serve as “radiological traps” due to their resemblance to hydronephrosis. This report details the case of a 38-year-old male whose. Initial ultrasonography demonstrated a large cystic lesion within the left renal sinus, raising suspicion for severe hydronephrosis. CT urography was subsequently performed to evaluate for obstructive uropathy. Delayed excretory phase imaging demonstrated contrast opacification within the lesion through a narrow communicating infundibulum, confirming the diagnosis of calyceal diverticulum. This case emphasizes that in the absence of an identifiable obstructive cause, delayed-phase imaging is mandatory to differentiate diverticula from obstructive uropathy, thereby preventing unnecessary surgical intervention.

## Introduction

A calyceal diverticulum is a rare developmental malformation where a portion of the renal collecting system fails to mature normally. Their prevalence is estimated between 0.21% and 0.6% in the general population [1,2]. Pathologically, these are urothelium-lined, nonsecretory cavities that communicate with the renal pelvis or calyx via a narrow infundibulum [3].

They are usually discovered incidentally but may be present with flank pain, infection, hematuria, or nephrolithiasis secondary to urinary stasis.

They are morphologically classified as type 1 and type 2 (Table 1).

**Table 1:** Differentiating points between type I and II calyceal diverticula.

Feature	Type I	Type II
Communication	Minor calyx	Major calyx/renal pelvis
Frequency	More common	Less common
Size	Smaller	Larger
Symptoms	Usually, asymptomatic	More symptomatic
Mimics hydronephrosis	Less often	More often
Stone formation	Possible	More common

Because these lesions appear as simple fluid collections on ultrasound, they are frequently mischaracterized as hydronephrosis or parapelvic cysts [4]. Definitive diagnosis requires contrast-enhanced imaging to visualize the slow transit of contrast through the narrow neck of the diverticulum.

## Case presentation

A 38-year-old man presented with a 3-month history of vague intermittent left flank pain. There was no history of fever, dysuria, hematuria, urinary tract infection, or nephrolithiasis. Laboratory investigations, including renal function tests and urinalysis, were within normal limits.

Initial transabdominal ultrasonography demonstrated a large well-defined cystic lesion within the left renal sinus, suggestive of gross hydronephrosis (however the narrow infundibulum visible on the Fig. 1A was missed on initial USG). No renal calculi or obvious obstructive lesion was identified. In view of suspected hydronephrosis without a definite obstructive cause, contrast-enhanced CT urography was performed for further evaluation.

**Fig. 1:**
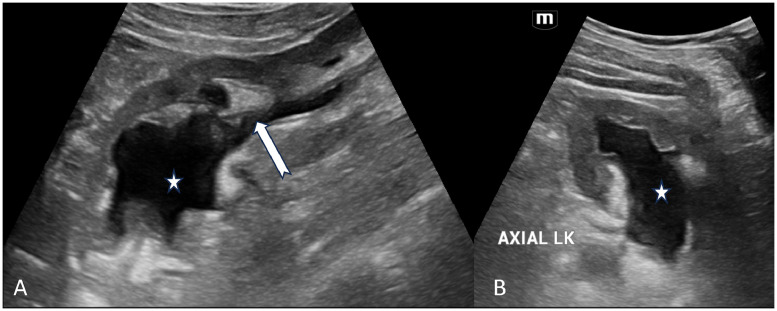
Longitudinal (A) and axial (B) grayscale ultrasonography images of the left kidney demonstrate a well-defined anechoic calyceal outpouching (star) arising from the upper pole calyx and communicating with the renal pelvis through a narrow infundibular neck (open arrow). The lesion shows smooth walls, posterior acoustic enhancement, and continuity with the collecting system without significant calyceal blunting or parenchymal thinning.

Early venous phase CT images revealed a well-defined cystic lesion adjacent to the left renal pelvis and upper pole major calyx, without evidence of ureteral dilatation. No obstructing calculus or mass lesion was identified (Fig. 2). Delayed excretory phase imaging obtained at 15 minutes demonstrated faint layering of excreted contrast material within the lesion. Additional delayed imaging acquired at 1 hour and 20 minutes (Fig. 3) showed more prominent opacification of the cavity confirming communication with the pelvicalyceal system and establishing the diagnosis of a calyceal diverticulum.

**Fig. 2:**
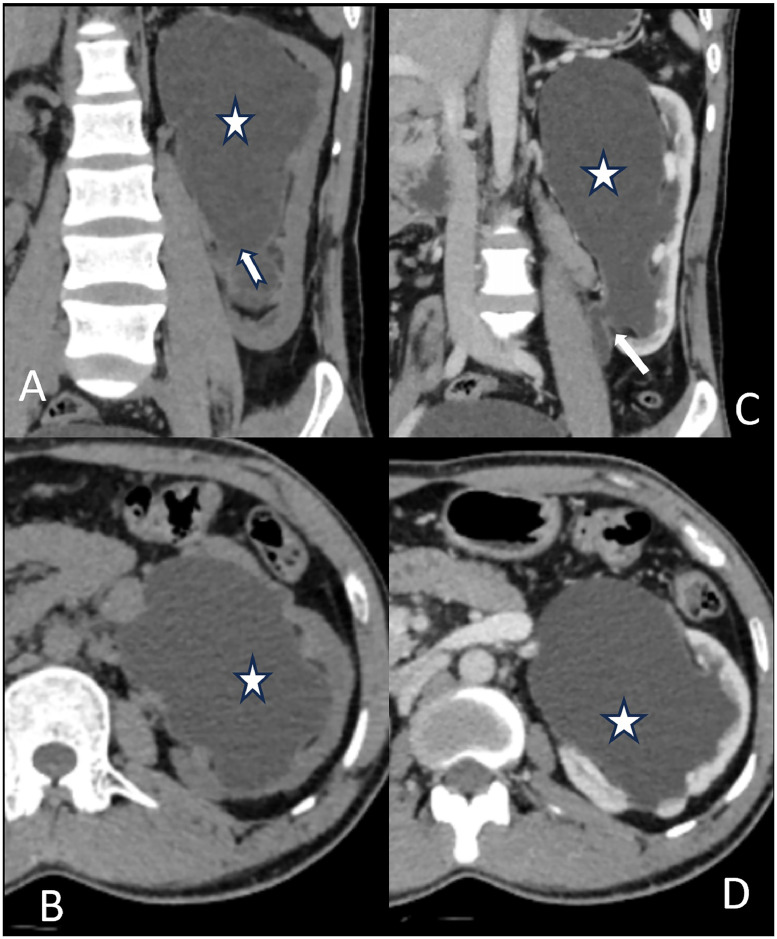
Noncontrast CT (A, B) and postcontrast CT (C, D) images of the abdomen in coronal (A, C) and axial (B, D) planes demonstrate a large well-defined hypodense calyceal outpouching involving the left kidney (star). The diverticulum communicates with the collecting system through a narrow infundibular channel (open arrow). The pelviureteric junction (white arrow) is displaced inferiorly due to the mass effect of the enlarged diverticulum. No enhancing solid component, mural nodularity, or calcification is identified. Mild compression and stretching of the adjacent renal parenchyma are noted without significant hydronephrosis or calyceal blunting. Postcontrast images show nonenhancing fluid attenuation contents with preserved enhancement of the surrounding renal parenchyma.

**Fig. 3:**
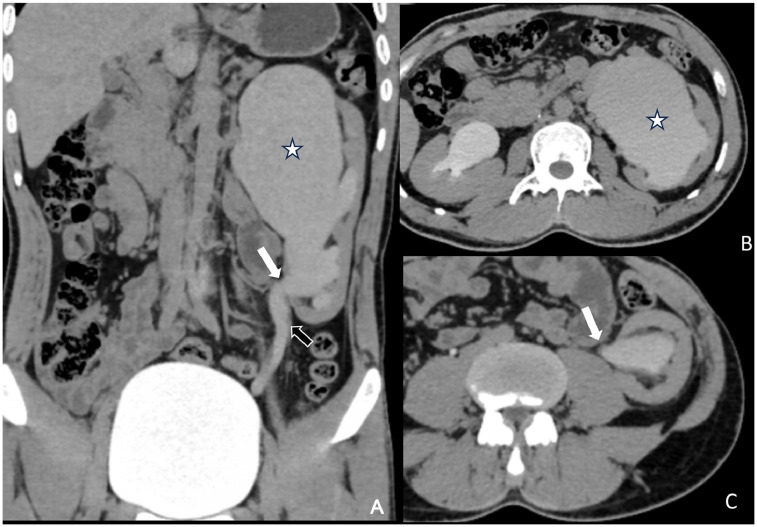
Delayed excretory phase CT images obtained at 1 hour 20 minutes in coronal (A) and axial (B, C) planes demonstrate persistent pooling and retention of contrast within the large left pelvicalyceal diverticulum/calyceal outpouching (star), confirming communication with the collecting system. The pelviureteric junction (white arrow) is displaced inferiorly, while the proximal ureter (black arrow) is seen coursing inferior to the diverticulum without significant upstream hydroureteronephrosis. Delayed opacification of the diverticulum through a narrow infundibular communication is noted, with prolonged contrast retention characteristic of a calyceal diverticulum.

Based on the large size and communication with the renal pelvis and upper pole major calyx, the lesion was consistent with a type II calyceal diverticulum.

There was no associated calyceal blunting, proximal ureteric dilatation, significant cortical thinning, or significant mass effect to suggest obstructive hydronephrosis. The patient was managed conservatively because symptoms were minimal and renal function was preserved.

## Discussion

Calyceal diverticula are uncommon benign outpouchings of the renal collecting system that communicate with a calyx or renal pelvis through a narrow infundibulum [5]. These lesions are classified into 2 types. Type I diverticula communicate with a minor calyx and are more common, whereas type II diverticula communicate directly with the renal pelvis or a major calyx and are usually larger and more symptomatic.

The clinical presentation is often nonspecific. Most patients are asymptomatic, although some may present flank pain, recurrent urinary tract infections, hematuria, or renal stone disease due to urinary stasis within the diverticulum [6]. Milk-of-calcium calculi may occasionally develop and can serve as an important diagnostic clue.

Radiologically, calyceal diverticula can closely mimic hydronephrosis or parapelvic cysts, particularly on ultrasonography. Hydronephrosis typically demonstrates dilatation of the collecting system associated with calyceal blunting and varying degrees of ureteric dilatation. In contrast, calyceal diverticula usually appear as isolated cystic lesions without diffuse collecting system dilatation or ureteric enlargement. Parapelvic cysts, although located within the renal sinus, do not communicate with the collecting system and therefore do not opacify on delayed excretory imaging (Table 2).

**Table 2:** Differentiating points concerning close differentials of calyceal diverticulum.

Feature	Calyceal diverticulum	Hydronephrosis	Parapelvic cyst
Communication with PCS	Present	Present	Absent
Delayed contrast filling	Yes	Immediate	No
Ureter dilatation	Usually, absent	Present	Absent
Calyceal blunting	Absent	Present	Absent
Stones	Common	Variable	Rare

CT urography with delayed excretory phase imaging remains the imaging modality of choice for diagnosis [7]. Delayed filling of contrast material within the lesion through a narrow communicating neck is considered the hallmark imaging feature. In the present case, delayed imaging was essential in differentiating the lesion from obstructive hydronephrosis and confirming the diagnosis.

Management depends on symptom severity and associated complications. Asymptomatic or minimally symptomatic patients are usually managed conservatively. Intervention may be considered in cases complicated by recurrent infection, persistent pain, or stone formation and may include endoscopic, percutaneous, or laparoscopic approaches [8].

This case highlights the importance of considering calyceal diverticulum in the differential diagnosis of cystic renal sinus lesions and emphasizes the value of delayed excretory phase imaging in avoiding unnecessary surgical intervention.

A brief review of previously reported cases and imaging findings of calyceal diverticulum is summarized in Table 3.

**Table 3:** A summary of brief review of previously reported cases and imaging findings of calyceal diverticulum.

Study	Clinical presentation	Imaging findings	Management
Timmons et al. [1]	Flank pain and hematuria	Intrarenal cystic cavity communicating with collecting system on IVU	Conservative management in asymptomatic patients
Wulfsohn [2]	Incidental finding and recurrent UTI	Delayed contrast filling of diverticular cavity	Observation or surgical management depending on symptoms
Kawashima et al. [3]	Flank pain	CT urography demonstrated delayed opacification through a narrow infundibulum	Conservative management
Okeke et al. [7]	Recurrent stones and pain	Calyceal diverticulum with associated calculi on CT imaging	Endoscopic/percutaneous intervention
Ananthapadmanabhan et al. [8]	Symptomatic calyceal diverticulum	CT with 3D modelling for surgical planning	Minimally invasive surgical treatment
Present case	Intermittent left flank pain	Lesion initially mimicked gross hydronephrosis on ultrasonography; delayed CT urography demonstrated communication with collecting system	Conservative management

## Conclusion

This case illustrates the potential for calyceal diverticula to simulate severe hydronephrosis on screening exams. When a patient presents with apparent renal collecting system dilatation but no identifiable obstruction, clinicians should prioritize delayed-phase CT imaging. This approach ensures a definitive diagnosis based on the passive retrograde flow of contrast into the diverticulum, confirming its connection to the renal pelvis [8]. Recognition of this entity is essential to establish the correct diagnosis and avoid unnecessary intervention.

### Refined learning points

• Calyceal diverticulum may mimic severe hydronephrosis on ultrasonography.
• A normal-caliber ureter in the absence of an obstructive lesion should raise suspicion for a hydronephrosis mimic.
• Delayed excretory phase CT urography is essential for demonstrating communication with the collecting system.
• Conservative management is appropriate for asymptomatic or minimally symptomatic patients.

## Patient consent

Written informed consent for publication of this case report and the accompanying images was obtained from the patient’s parent/legal guardian.
